# The benefits of Cu *K*β radiation for the single-crystal X-ray structure determination of crystalline sponges

**DOI:** 10.1107/S2052252522002147

**Published:** 2022-03-10

**Authors:** Florian Meurer, Carolina von Essen, Clemens Kühn, Horst Puschmann, Michael Bodensteiner

**Affiliations:** a University of Regensburg, Universitätsstrasse 31, Regensburg, 93053 Bayern, Germany; b Merck Innovation Center, Merck KGaA, Frankfurter Strasse 250, Darmstadt, 64293 Hessen, Germany; c OlexSys Ltd, Chemistry Department, Durham University, Durham DH1 3LE, United Kingdom

**Keywords:** crystalline sponge, copper *K*β radiation, metal–organic frameworks, X-ray analysis

## Abstract

The crystalline sponge method facilitates the X-ray structure determination of samples that do not crystallize or are too sparsely available to afford viable crystallization. By including these materials in a metal–organic framework, the structure of the guest molecules can be determined. Some of the inherent difficulties of this method are discussed and the use of Cu *K*β radiation is presented as a simple and effective means to improve the quality of the diffraction data that can be obtained from a sponge crystal.

## Abstract 2

Copper *K*β radiation offers some advantages over its *K*α counterpart that prove very useful for determining crystal structures in metal–organic framework host–guest systems.

## 1. Introduction

The crystalline sponge (CS) method, as introduced in 2013 by Fujita *et al.*, can facilitate the X-ray diffraction (XRD) structure determination of certain challenging samples, particularly those that are only available in minute quantities, and where single crystals cannot be obtained. The underlying principle is that the analyte does not itself need to be crystalline, as long as it can be absorbed in a crystalline framework in a sufficiently ordered manner that its structure can be determined as a guest molecule (Inokuma *et al.*, 2013 ▸; Stallforth & Clardy, 2013 ▸). The linking of late first-row transition metal halides such as Zn*X*_2_ (*X* = Cl, I) by organic, nucleophilic compounds results in a three-dimensional metal–organic framework (MOF), the host or sponge. These Fujita-type frameworks feature cavities large enough to be accessible by solvents and other small organic molecules. Functional groups such as halides bound to the metal centre or nitro­gen atoms within the linker molecule can act as hydrogen-bond acceptors and in the presence of aromatic systems allow for π–π interactions. By this, they direct the guest analytes within the framework into a particular orientation: an analyte molecule can be detected as part of the crystal structure by single-crystal X-ray diffraction (SCXRD) (Hoshino *et al.*, 2016 ▸; Hayes *et al.*, 2016 ▸). Anything that can be included in the sponge can be analysed, including solutes in highly dilute solutions, gases and oils, which could not be structurally characterized by X-rays any other way (Inokuma *et al.*, 2013 ▸).

Despite the immense potential of the method and recent improvements and successes (Hoshino *et al.*, 2016 ▸; Hayes *et al.*, 2016 ▸; Ramadhar *et al.*, 2015*a* ▸,*b* ▸; Easun *et al.*, 2017 ▸; Duplan *et al.*, 2016 ▸; Cuenca *et al.*, 2016 ▸; Cardenal & Ramadhar, 2021 ▸; Bosch *et al.*, 2014 ▸; Goh *et al.*, 2018 ▸; Inokuma *et al.*, 2016 ▸, 2014 ▸; Kersten *et al.*, 2017 ▸; Lee *et al.*, 2017 ▸; Li *et al.*, 2019 ▸; Matsuda *et al.*, 2016 ▸; Mitsuhashi *et al.*, 2018 ▸; Morishita *et al.*, 2020 ▸; Morita *et al.*, 2020 ▸; Rissanen, 2017 ▸; Vinogradova *et al.*, 2014 ▸; Wada *et al.*, 2018 ▸; Waldhart *et al.*, 2016 ▸; Yoshioka *et al.*, 2016 ▸; Yuan *et al.*, 2019 ▸; Zigon *et al.*, 2015 ▸; de Poel *et al.*, 2019 ▸; Urban *et al.*, 2016 ▸; Sakurai *et al.*, 2017 ▸) it is still far from being the universal way to determine the structure of troublesome compounds, because the method still faces some serious challenges: the time-consuming process of analysis is highly dependent on the analytes and must be carried out in separate batches for each sample. The obtained crystal structures are often crystallographically challenging because of the nature of the particular host–guest interactions (Cardenal & Ramadhar, 2021 ▸; Hoshino *et al.*, 2016 ▸; Zigon *et al.*, 2021 ▸). If this interaction between host and guest is too strong, diffusion into the cavities of the sponge is drastically hindered, if not impossible. If, on the other hand, the interaction is too weak, the analyte in the sponge may substantially lack orientation. As a result, in the worst case, the compound of interest is no longer detectable in the diffraction experiment. In most cases – between these two extremes – more or less severe disorder is encountered. Additionally, twinning and radiation damage both pose serious issues to a successful structural determination of the analyte.

Overcoming the many issued faced by the CS method is the subject of the current work: in 2016 Fujita *et al.* reported optimized parameters for the preparation of host crystals, soaking procedure and X-ray experimental conditions, as well as on a modification enabling reliable absolute structure determination (Hoshino *et al.*, 2016 ▸; Urban *et al.*, 2016 ▸). A new method for the optimization of process parameters for faster guest introduction was recently reported (Rosenberger *et al.*, 2021 ▸). Many further investigations including, for example, *f*-block elements as building blocks for crystalline sponges also contributed to the improvement of the CS method (de Poel *et al.*, 2016 ▸). For a more comprehensive overview, the recent reviews by Ramadhar, Cardenal and the Fujita group are recommended (Cardenal & Ramadhar, 2021 ▸; Zigon *et al.*, 2021 ▸).

From a crystallographic point of view, recent developments in diffractometer technology and software are also of great benefit to the CS method. Modern X-ray sources and detectors lead to ever-increasing data quality of the X-ray diffraction pattern collected: higher intensity, higher redundancy and higher resolution all help in dealing with the problems inherent to MOF structures (Hoshino *et al.*, 2016 ▸). However, Cu *K*α radiation is limited in resolution to about 0.8 Å. Absorption is another issue of this rather low X-ray energy, especially in case of iodine-containing sponge crystals. Furthermore, the Cu *K*α_1,2_ splitting is a shortcoming since it aggravates the disappearance of the reflections into the background noise.

Herein, we investigate the advantages that the use of Cu *K*β radiation can bring to structures of these (and other) MOFs. Cu *K*β is the second line in the copper emission spectrum and is accessible by replacing the mirror optics of a standard copper X-ray source. Cu *K*β is not traditionally used for SCXRD experiments, since its raw intensity is only around 1/8 of the commonly used Cu *K*α and is typically removed by monochromatization (Thompson *et al.*, 2009 ▸).

Despite the lower intensity, especially the lack of splitting of the reflection signal at high resolution is a good reason to use Cu *K*β, and this has been explored mostly for powder XRD experiments (Otto, 2018 ▸). We have started to explore the use of Cu *K*β for SCXRD experiments and found strong advantages in general and particularly for problematic compounds (Mayr, 2018 ▸; Marquardt *et al.*, 2016 ▸). Due to the higher resolution of the shorter wavelength, 36% more unique reflections are obtained with Cu *K*β. This significantly improves the electron density map and is beneficial for modelling atomic displacement parameters and disorder (Bennett, 2010 ▸). Absorption is also significantly reduced for almost every element for Cu *K*β compared with Cu *K*α, with nickel being the most notable exception as it is known as a cost-efficient filter for Cu *K*β (Seltzer, 1995 ▸; Linstrom, 1997 ▸). This again can be largely attributed to the shorter wavelength of Cu *K*β. These advantages generally improve the expected measurement times for comparable signal-to-noise ratios up to only 2.5× the time required for a standard Cu *K*α experiment (Mayr, 2018 ▸). This ratio is highly dependent on the particular compound and often the experiment times are much more comparable, especially when heavy, highly absorbing elements are present.

In summary, the differences between Cu *K*α and Cu *K*β are mainly in favour of the latter, especially when interfering disorder with limited resolution and strongly absorbing elements are present. This is generally the case for the structural elucidation of MOFs, but especially for the application of the CS method, where the exact configuration and connectivity of an unknown guest molecule is of interest. Cu *K*β has a lower absorption for all the typical elements present in crystalline sponges. Background noise, absorption artefacts and radiation damage are all expected to be less significant when measuring with Cu *K*β radiation.

A more general and comprehensive paper on the advantages and differences of Cu *K*β compared with both Cu *K*α and Mo *K*α is currently in preparation.

## 2. Results and discussion

To gain insight into the benefits of the different wavelengths, three comparison experiments (Table 1 ▸) have been performed with Cu *K*α_1,2_ and Cu *K*β_1,3_ radiation to a total of six experiments. Sponge crystals were elucidated containing either only the soaking solvent cyclo­hexane or Me-Daidzein [7-Meth­oxy-3-(4-meth­oxy­phenyl)­chromen-4-on, Me-Daid] as testing analyte. Measurements were taken on a selection of the two most widely used Fujita-type sponge crystals: [(ZnCl_2_)_3_·tpt_2_]_*n*_ [tpt = 2,4,6-*tris*(4-pyridyl)-1,3,5-trazine] and [(ZnI_2_)_3_·tpt_2_]_*n*_. The crystalline sponges for this study were provided by Merck KGaA via the ‘Crystal-Do’ project of the Merck Innovation Center.

A fresh crystal from the same preparation batch was used for each subsequent measurement to minimize potential damage to the crystal through radiation and repeated handling. The storage of the crystal during the optic replacement and the following recalibration of the diffractometer made it impossible to keep the crystal inside the low-temperature thermostat of the diffractometer.

Cu *K*β experiments were performed to a maximum resolution of 0.72 Å, with the comparison Cu *K*α experiments being performed up to 0.80 Å. Every data collection took place on the same diffractometer (micro-focus copper anode tube and CCD detector) at a temperature of 100 K with interchanged optics for the respective wavelength. Further experimental setup and processing details are given in the supporting information. To allow for a direct comparison regarding signal-to-noise ratios and reliability factors, we calculated these to a resolution of 0.80 Å. It is reported that lower-resolution data generally result in a lower *R*_1_ value, as *R*_1_ favours stronger data (Arnberg *et al.*, 1979 ▸). Unfortunately, this fact often shifts the attention away from weaker reflections. These are essential for a well defined model (Arnberg *et al.*, 1979 ▸). Hirshfeld & Rabinovich (1973 ▸) stated that there is no physical basis on which to dismiss reflections based on their average signal-to-noise ratio, *e.g.* within a certain regime of resolution.

**Figure 1:**
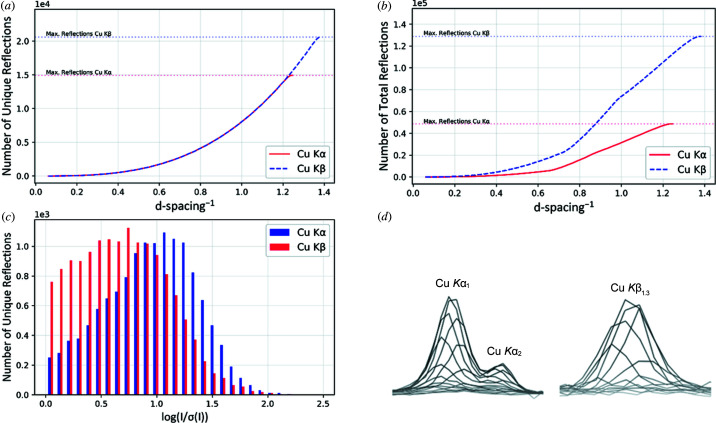
Increase in number of (*a*) unique and (*b*) total data with increasing resolution and (*c*) distribution of reflections versus intensity of measurements 1a and 1b. Respective plots for all measurements can be found in the supporting information. (*d*) Side profile of an arbitrarily chosen reflection (14 14 17) from experiment 1 on the detector at a resolution of 0.81 Å for Cu *K*α and Cu *K*β radiation in line representation.

Omission of all weak reflections within a resolution shell discards underestimated reflections more readily than it discards those overestimated. As intensity errors are expected to be normally distributed, this harms statistical integrity and has a significant negative impact on atomic parameters and their standard deviations (Hirshfeld & Rabinovich, 1973 ▸). The data measured for this work are an example for this case: experiments using Cu *K*β resulted in a lower average signal-to-noise ratio especially because of the weak, high-resolution data [Fig. 1 ▸(*c*)]. Inclusion of this data, on the one hand, allows for the modelling of better structures. On the other hand, cutting the datasets and omitting this high-resolution information improves the quality indicators *R*_1_ and *wR*_2_. Therefore, we decided on the quality of the structures not only on these indicators, but also on the C—C bond precision and residual electron density.

In Table 1 ▸ selected measurement parameters of 1, 2 and 3 are shown. The longest comparison measurement time for Cu *K*β is observed for the chloride species (1a and 1b). Here, the measurement takes roughly twice the time needed for the respective Cu *K*α experiment. On the contrary, the measurement times are below a ratio of two for measurements 2a and 2b and nearly identical for the measurements 3a and 3b. In these last comparison measurements, identical experiment times resulted in similar signal-to-noise ratios, even though a crystal of half the size was measured in the Cu *K*β experiment. In the measurements 2a and 2b, twinning in the case of the Cu *K*β experiment caused complications; nonetheless, a similar signal-to-noise ratio was obtained.

The observed signal-to-noise ratios do not at all resemble the ratio of raw intensities of both wavelengths of 1:8 in favour of Cu *K*α. This is due to the lower elemental absorption for each element present (C, N, O, Zn, Cl or I). This leads to lower absorption, thus higher relative intensities, and we generally observed lower noise for Cu *K*β relative to the Cu *K*α experiments. Consequently, the absorption coefficients are significantly lower when using Cu *K*β: throughout the measurements a 25% lower absorption coefficient was calculated. Especially for the sponge crystals containing iodine, a decrease from ∼20 mm^−1^ in absorption coefficient for Cu *K*α to ∼15 mm^−1^ for Cu *K*β was observed for the data collection which is also beneficial for the experiment times. Cu *K*β performace is enhanced when stronger absorbing elements are present.

Another advantage of lower absorption is the less-frequent occurrence of absorption-related processes such as radiation damage. Since absorption is associated with strong electronic excitation, the spontaneous formation of radicals can lead to local crystal defects, further decreasing the overall quality of the data (Christensen *et al.*, 2019 ▸; Garman, 2010 ▸). However, to what extent this was the case in the experiments performed for this work can only be described qualitatively: two crystals with the composition of 3 were both exposed for 2 h at 100 K in a static position at each wavelength. Only the crystal irradiated with Cu *K*α underwent a significant change in colour from colourless to a dark green. In contrast, the crystal exposed to Cu *K*β remained colourless.

In terms of reflection statistics, the shorter Cu *K*β wavelength gave 23 to 38% additional unique data and up to 163% additional total data. Figs. 1 ▸(*a*)–1(*c*) show that these additional data for measurement 1b originate from the weak, high-resolution regime, mainly between 0.80 and 0.72 Å resolution. In this domain, the crystals visibly approached their physical resolution limit of diffraction.

Data with a resolution higher than 0.80 Å cannot be obtained with Cu *K*α. Although these high-resolution reflections are of low intensity for Cu *K*β, they contribute strongly to the overall model and to the standard uncertainties. Thus, these reflections helped, for example, to model disorder, where this disorder was not obvious from lower-resolution data and benefited atomic parameters such as bond distances.

For the iodide species, the additional data were also obtained in the high signal-to-noise ratio range. We observed more and surprisingly stronger reflections for Cu *K*β than for Cu *K*α (Figs. S2 and S3 of the supporting information). Notably, in both cases, additional unique data for Cu *K*β are collected from about 0.80 Å to full resolution, outside the physical limit of Cu *K*α [Fig. 1 ▸(*a*)]. The additional total data are evenly obtained over the full range of resolution [Fig. 1 ▸(*b*)]. Since more total data can be obtained in the Cu *K*β experiments, the longer measurements are used more efficiently in terms of time. In combination with the lower absorption already mentioned, this also helps to avoid radiation damage, as more reflections are obtained with a fixed dose of radiation, and is beneficial for obtaining sufficient redundancy.

Furthermore, the arbitrarily chosen high-resolution reflection in measurement 1 [Fig. 1 ▸ (*d*)] shows that peak-splitting for the respective Cu *K*α_1,2_ lines is pronounced in the Cu *K*α experiment. In the corresponding Cu *K*β_1,3_ experiment, no peak-splitting was observed. This proves to be beneficial to the data processing, where integration masks do not have to take this resolution-dependent peak-splitting into account. The signal-to-noise ratio of a single Cu *K*β Gaussian profile shows relative improvement compared with the partially separated 2:1 profile of Cu *K*α. All these advantages for Cu *K*β result in better quality parameters and models.

Throughout the measurements, the lowest *R*_1_ and *wR*_2_ values were obtained in the Cu *K*β datasets. There is an overall better agreement of the observed and calculated structure factors, including atomic displacement parameters as well as atomic occupancies in the data collected with Cu *K*β. This is despite *R*_1_ favouring strong data and despite prominent twinning being present in the case of the Cu *K*β crystal in measurement 2b. It is also only in this pair of comparison measurements where the Cu *K*α experiment has a better C—C bond precision (Cu *K*α: 0.025 Å, Cu Kβ: 0.031 Å). In the other experiments, the models obtained with Cu *K*β were significantly more accurate (1 – Cu *K*α: 0.010 Å, Cu *K*β: 0.006 Å; 3 – Cu *K*α: 0.027 Å, Cu *K*β: 0.019 Å). This was the case even though generally fewer restraints had to be applied to the models using Cu *K*β.

**Figure 2:**
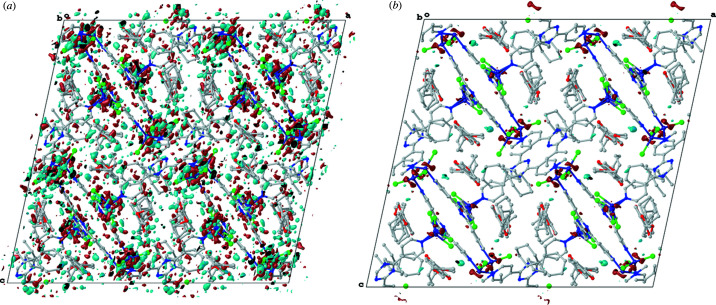
Residual electron density map (iso value: 0.5 e Å^−3^) for measurement 1: (*a*) Cu *K*α and (*b*) Cu *K*β measurements with the sponge scaffold, analyte and solvent in ball and stick representation along the *b* direction. Hydrogen atoms have been omitted for clarity.

The more accurate atomic parameters as well as less absorption result in an astonishing difference in the residual density maps (Figs. 2 ▸, S4 and S5). The models obtained with Cu *K*β show a much cleaner map inside the cavity of the sponge scaffolds and significantly less absorption artefacts around the bound halides. Most prominently, Cu *K*β shows some diffuse residual density around the transition metal. This can be attributed to the independent atom model (IAM) used in crystallography, which does not account for non-spherical density distribution. This aspherical density would be expected when describing transition metal bonding and signals rather the limits of the IAM (Capelli *et al.*, 2014 ▸; Kleemiss *et al.*, 2021 ▸).

The cleaner residual electron density maps can be attributed to the fact that the additional data for Cu *K*β led to an improved assignment of the obtained electron density. This results in more accurate and freely refined occupancies (Fig. S6), fewer restraints on atomic parameters, and no constraints.

For measurement 1, disordered solvent positions could be located from the residual density map of the Cu *K*β datasets, which were not recognisable from the Cu *K*α data. Assignment of remarkably low 0.16 units of solvent was possible at a position shared with another 0.30 units of solvent and 0.50 units of testing analyte Me-Daid. These could be refined without constraints on their occupational parameters. In every Cu *K*α measurement, the typically disordered Zn*X*_2_ unit had to be refined with constrained atomic displacement parameters. For comparison, only soft atomic displacement parameter restraints had to be applied for the respective disorder in every corresponding Cu *K*β dataset.

Effects of this kind lead to a certain ‘auto-acceleration’. A more defined electron density map yields better modelled atomic parameters, which then allows for better resolution of the residual density map. This is the main reason why the datasets employing Cu *K*β yield superior models.

## 3. Conclusions

The use of Cu *K*β radiation for the determination of crystalline sponges has unquestionably resulted in better structures compared with using the ‘standard’ Cu *K*α radiation. The effect of the lower output intensity of Cu *K*β compared with Cu *K*α is easily compensated by yielding more data (at the same resolution), higher overall resolution, less absorption and the absence of high-resolution peak-splitting. The best reliability parameters were obtained in the Cu *K*β experiments for every comparison experiment performed. Bond lengths were determined more accurately when using Cu *K*β. The allocation and refinement of solvent positions is better, and more positions can be unambiguously determined in the experiments performed using Cu *K*β. The improved models allow for a better localization in the Fourier map, leading to fewer restraints and constraints on atomic parameters. Free refinement of occupancies, of both the sponge host and the guest molecules, is achieved for the Cu *K*β data. Most significantly, this resulted in cleaner residual electron density maps for all Cu *K*β experiments.

In total, this will give a decisive advantage for the CS method, where unknown, mostly organic, analytes must be carefully deduced from the residual electron density map. Absorption artefacts around halides, which are complicated to deal with, are significantly reduced by the shorter wavelength. Disorder and partially occupied sites can be modelled better against data obtained with Cu *K*β. Theoretically, one could expect these advantages to be even more pronounced when Mo *K*α radiation is used, as it inherits an even higher possible resolution. However, because of their diffuse content and large unit cells, crystalline sponges and other MOFs tend to have a restricting physical diffraction limit. Additionally, the output intensity of an Mo source is lower than for both copper wavelengths. Zinc, the common heavy element in all Fujita-type sponge crystals, absorbs the least for Cu *K*β, even compared with Mo *K*α, as Zn is in the ‘sweet spot’ of absorption for Cu *K*β. These considerations for a comparison to Mo *K*α as well as the advantages of Cu *K*β for absolute structure determination of chiral guests will be subjects of our future studies.

For many problems concerning the crystallography of sponge crystals, Cu *K*β radiation has shown substantial improvements. These might be subtle, but game-changing enhancements that a structure may need to become publishable.

## 4. Related literature

For further literature related to the supporting information, see Bourhis *et al.* (2015 ▸), Sheldrick (2015*a* ▸,*b* ▸) and Spedicato *et al.* (2003 ▸).

## Supplementary Material

Crystal structure: contains datablock(s) alpha_me-daid_zncl2, beta_me-daid_zncl2, alpha_me-daid_zni2, beta_me-daid_zni2, alpha_cyc_zni2, beta_cyc_zni2. DOI: 10.1107/S2052252522002147/lt5046sup1.cif

Supporting information file. DOI: 10.1107/S2052252522002147/lt5046sup2.pdf

CCDC references: 2091650, 2091651, 2091652, 2091653, 2091654, 2091655

## Figures and Tables

**Table 1:** Performed measurements and selected experiment parameters

No.	Species	Analyte	Parameters					
			Radiation type	Experiment time	*R*_1_ †, *wR*_2_ † (%)	*I*/σ(*I*)†	Crystal volume (10^−2^ mm^−3^)	Max peak, hole† (eÅ^−3^)
1a	Chloride	Me-Daid	Cu *K*α	45 h 24 min	8.6, 23.5	20.3	0.108	1.22, −0.69
1b	Chloride	Me-Daid	Cu *K*β	89 h 8 min	5.9, 18.4	14.4	0.0588	0.65, −0.81
2a	Iodide	Me-Daid	Cu *K*α	24 h 55 min	12.3, 35.6	14.4	0.570	1.73, −2.18
2b	Iodide	Me-Daid	Cu Kβ	40 h 1 min	11.0, 35.0	14.1	0.149	1.20, −1.90
3a	Iodide	Cyc	Cu Kα	13 h 24 min	9.42, 25.1	16.3	0.187	3.21, −2.38
3b	Iodide	Cyc	Cu Kβ	13 h 30 min	7.7, 15.9	13.6	0.112	1.22, −1.36

† Calculated to a resolution of 0.80 Å, Cu *K*β data was measured to 0.72 Å.
