# Supplementary material for: The benefits of Cu Kβ radiation for the single-crystal X-ray structure determination of crystalline sponges
Source: IUCrJ. 2022 Mar 10;9(Pt 3):349–54. doi: 10.1107/S2052252522002147 (PMC9067116; doi:10.1107/S2052252522002147)
Supplement: Supplementary file 2 [file m-09-00349-sup2.pdf]

IUCrJ

Volume 9 (2022)

Supporting information for article:

The benefits of Cu Kβ radiation for the single crystal X-ray structure determination of crystalline sponges

Florian Meurer, Carolina von Essen, Clemens Kühn, Horst Puschmann and Michael Bodensteiner

S1. Experimental procedures

The crystalline sponges $[(ZnCl_2)_3\cdot(\textbf{tpt})_2\cdot x(\text{cyclohexane})]_n$ and $[(ZnI_2)_3\cdot(\textbf{tpt})_2\cdot x(\text{cyclohexane})]_n$ were prepared following the reported procedures. (Fujita *et al.*, 2016) Crystals for measurements **3** were prepared in absence of an analyte and contain only the soaking solvent cyclohexane.

S1.1. Preparation of inclusion compounds for 1 and 2:

A single crystal of the porous complex $[(ZnX_2)_3\cdot(\textbf{tpt})_2\cdot x(\text{cyclohexane})]_n$ (X=I, Cl) was transferred with 50 µl of solvent to a sample vial. 1 µl of 7-Methoxy-3-(4-methoxyphenyl)chromen-4-on dissolved in dichloromethane (1 mg/ml) was pipetted into the sample vial. The vial was closed with a screw cap with septum seal and pierced with a syringe needle for slow evaporation of the solvent using an incubator at 50 °C for 1 day.

S1.2. Measurement and refinement procedure

All crystals were mounted onto a Rigaku Oxford Diffraction SuperNova diffractometer. The data were collected on a TitanS2 CCD detector. Data reduction, scaling and absorption correction were performed using CrysAlisPro (Rigaku Oxford Diffraction, 2020). Analytical and empirical absorption correction using spherical harmonics, implemented in SCALE3 ABSPACK scaling algorithm was carried out. (Spedicato *et al.*, 2003) The structure solution and refinement were conducted using ShelXT (Sheldrick, 2015b) and ShelXL (Sheldrick, 2015a) inside Olex2. (Bourhis *et al.*, 2015) All non-Hydrogen atoms were refined anisotropically. Hydrogen atom positions were calculated geometrically and refined using the riding model.

Disordered and partially occupied solvent molecules were modelled with restraints on occupational and displacement parameters. Especially the measurements with Cu Kα radiation resulted in models that also required displacement parameter constraints. In measurements **2 a** and **b** a solvent masking was applied. Crystal **2b** revealed non-merohedral twinning. Further details can be found in the respective crystallographic information file.

S2. Crystallographic data

Table S1 Crystallographic data for Cu Kα measurements

Compound	$[(ZnCl_2)_3(tpt)_2Me\text{-}Daid]_n$	$[(ZnI_2)_3(tpt)_2Me\text{-}Daid]_n$	$[(ZnI_2)_3(tpt)_2x(C_6H_{12})]_n$
Identifier	**1a**	**2a**	**3a**
CCDC Nr.	2091650	2091651	2091653
Empirical formula	$C_{68.39}H_{67.65}Cl_6N_{12}O_4Zn_3$	$C_{54.5}H_{43}I_6N_{12}O_4Zn_3$	$C_{63}H_{78}I_6N_{12}Zn_3$
Formula weight / g·mol^{-1}	1530.54	1887.52	1960.88
Crystal size / mm^{-3}	0.12×0.10×0.09	0.16×0.13×0.09	0.25×0.19×0.12

Temperature / K	100.00(10)	100.00(10)	100(2)
a / Å	33.3132(5)	34.4961(13)	34.5713(5)
b / Å	14.4948(2)	14.9830(6)	15.1587(3)
c / Å	31.7714(5)	31.0198(12)	29.5198(5)
α / °	90	90	90
β / °	102.245(2)	101.970(4)	100.6492(14)
γ / °	90	90	90
V / $Å^{-3}$	14992.4(4)	15684.1(11)	15203.5(4)
ρ / g·cm^{-3}	1.356	1.599	1.713
μ / mm^{-1}	3.511	19.985	20.567
θ-range	2.715 - 73.744	3.653 - 73.991	4.245 - 73.919
Total reflections	47323	28426	43747
Unique reflections	14931	14938	15078
Completeness	0.9990	0.9770	0.9940
R_1, wR_2, R_{int} / %	8.62, 23.5, 4.22	12.3, 34.2, 5.77	9.42, 25.1, 7.52
GooF	1.088	1.066	1.091
Largest diff. peak, hole / e· $Å^{-3}$	1.215, -0.687	1.727, -2.174	3.215, -2.379
No. of restraints	296	200	320
No. of parameters	1290	753	707
$I/\sigma(I)$	20.3	14.4	16.3
Indices range	41 ≥ h ≥ -40	23 ≥ h ≥ -40	43 ≥ h ≥ -40
	17 ≥ k ≥ -17	17 ≥ k ≥ -18	18 ≥ k ≥ -17
	39 ≥ l ≥ -37	38 ≥ l ≥ -35	36 ≥ l ≥ -35
Exposure time (inner, outer) / s	20, 80	20, 100	7.5, 30

Table S2 Crystallographic data for Cu Kβ measurements

Compound	$[(ZnCl_2)_3(tpt)_2Me\text{-}Daid]_n$	$[(ZnI_2)_3(tpt)_2Me\text{-}Daid]_n$	$[(ZnI_2)_3(tpt)_2x(C_6H_{12})]_n$
Identifier	**1b**	**2b (twinned)**	**3b**
CCDC Nr.	2091652	2091655	2091654
Empirical formula	$C_{68.63}H_{68.94}Cl_6N_{12}O_4Zn_3$	$C_{55}H_{42}I_6N_{12}O_4Zn_3$	$C_{64.72}H_{81.45}I_6N_{12}Zn_3$
Formula weight / g·mol^{-1}	1534.65	1892.51	1984.86
Crystal size / mm^{-3}	0.14×0.07×0.06	0.19×0.08×0.07	0.15×0.11×0.09
Temperature / K	100.01(10)	100.01(10)	100(2)

a / Å	33.3060(13)	34.4862(8)	34.5950(6)
b / Å	14.4880(4)	15.0052(4)	15.1725(3)
c / Å	31.8026(11)	31.1250(7)	29.5350(4)
α / °	90	90	90
β / °	102.247(4)	101.958(2)	100.6467(15)
γ / °	90	90	90
V / $Å^{-3}$	14996.7(9)	15756.8(7)	15235.8(5)
ρ / g·cm^{-3}	1.359	1.596	1.731
μ / mm^{-1}	2.497	15.035	15.175
θ-range	2.452 - 75.676	3.750 - 75.309	3.830 - 74.433
Total reflections	125624	49957	44964
Unique reflections	20592	21071	18530
Completeness	1.0000	0.9960	0.9620
R_1, wR_2, R_{int}	7.38, 25.4, 10.7	12.3, 42.2, -**	8.67, 17.9, 6.02
R_1, wR_2, R_{int} *	5.88, 18.4, 10.3	11.7, 37.11, -**	7.66, 15.9, 5.89
GooF	1.047	1.164	1.164
Largest diff. peak, hole / e· $Å^{-3}$	0.657, -1.120	1.488, -2.565	1.261, -1.948
Largest diff. peak, hole / e· $Å^{-3}$ *	0.648, -0.812	1.215, -1.949	1.224, -1.363
No. of restraints	390	315	383
No. of parameters	1285	813	930
I/σ(I)	12.6	13.8	12.7
I/σ(I) *	14.4	14.1	13.6
Indices range	45 ≥ h ≥ -45	47 ≥ h ≥ -41	45 ≥ h ≥ -47
	19 ≥ k ≥ -18	20 ≥ k ≥ -20	19 ≥ k ≥ -11
	43 ≥ l ≥ -43	43 ≥ l ≥ -39	39 ≥ l ≥ -40
Exposure time (inner, outer)	30, 90	30, 90	12.5, 50

* for data up to d = 0.8 Å; ** no meaningful R_{int} for reason of twinning

S3. Supplementing figures

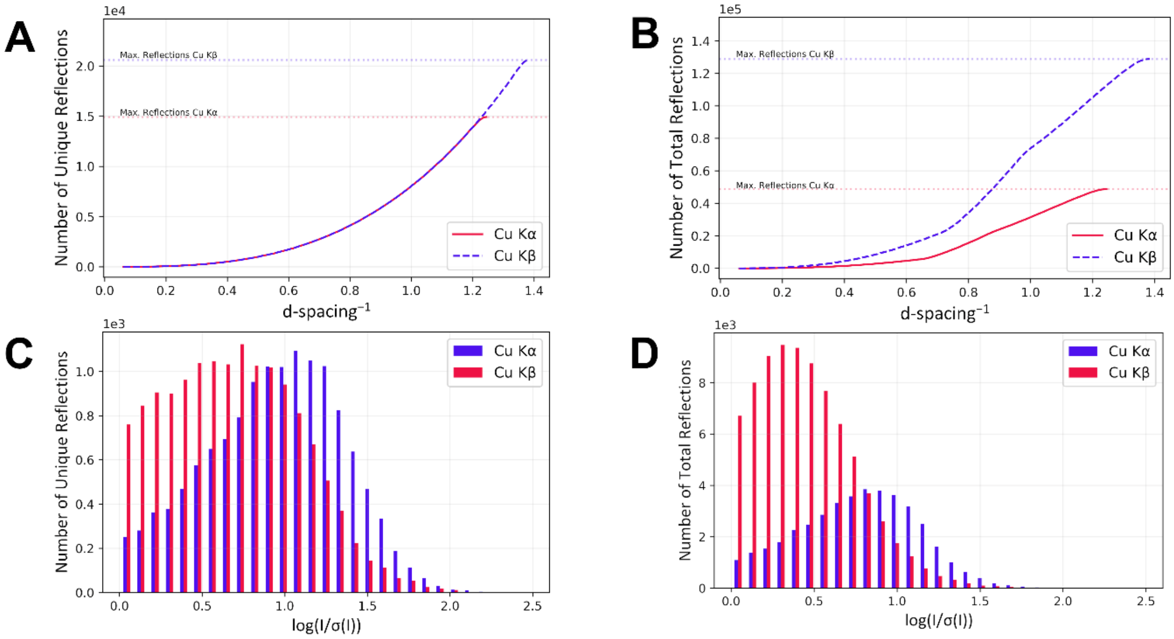

Figure S1 Reflection distribution by intensity and resolution for unique (**A**, **C**) and total (**B**, **D**) data for measurements **1 a** & **b**

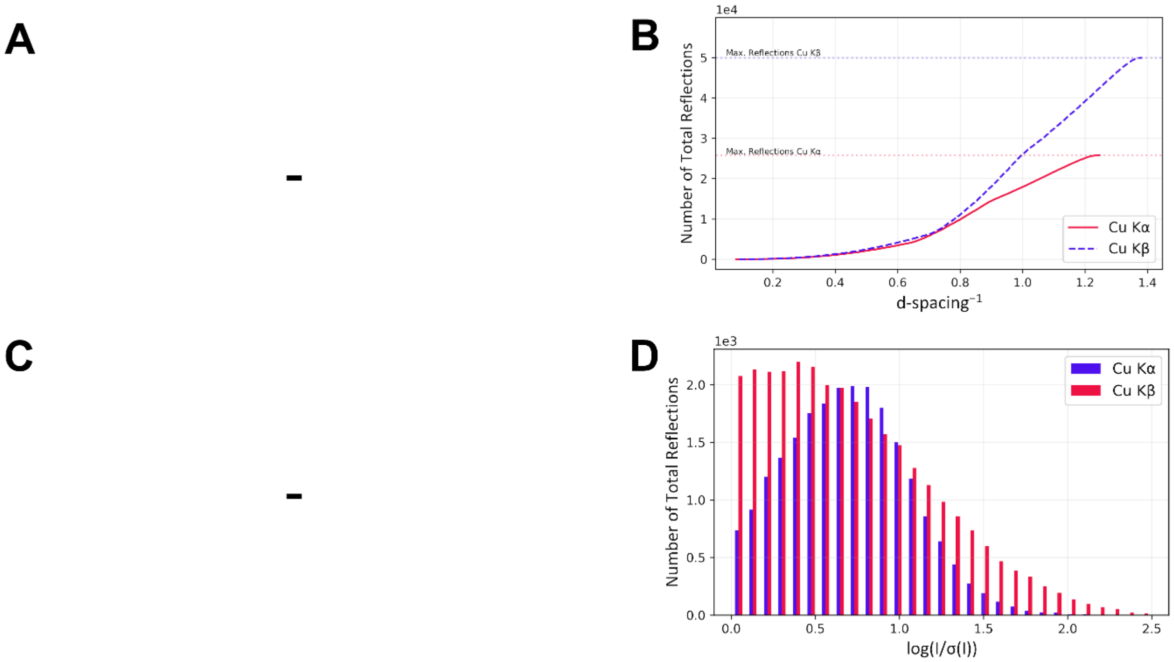

Figure S2 Reflection distribution by intensity and resolution for total (**B**, **D**) data for measurements **2 a** & **b**. Please note that as data set **2b** was twinned, a comparison of unique data is not meaningful.

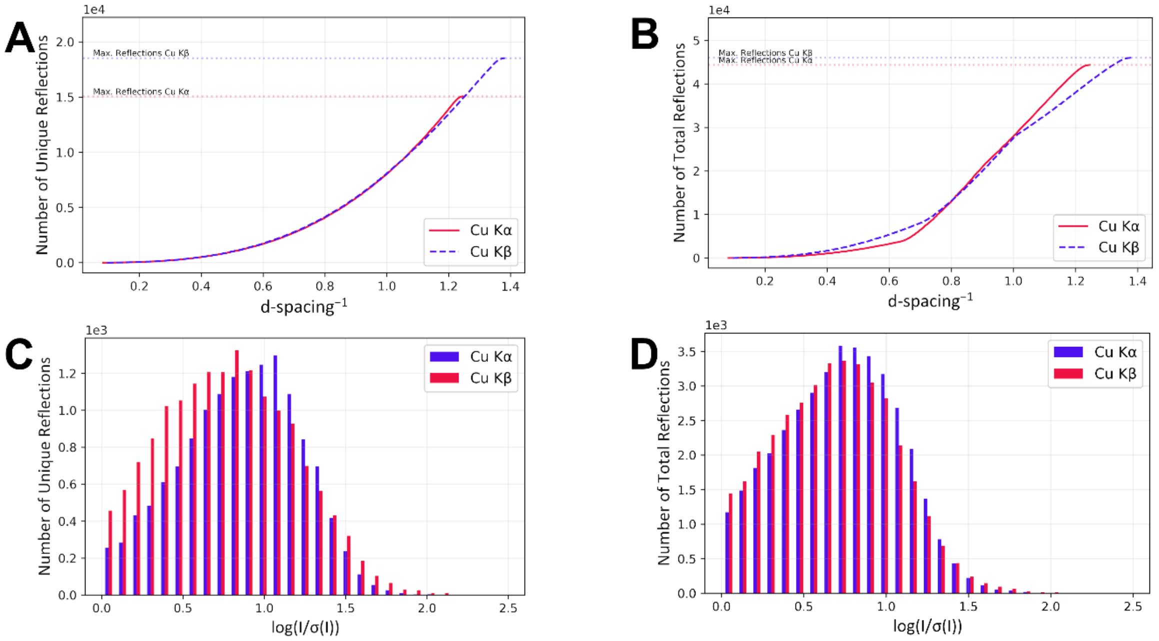

Figure S3 Reflection distribution by intensity and resolution for unique (**A**, **C**) and total (**B**, **D**) data for measurements **3 a** & **b**

S4. Comparison of residual electron density maps

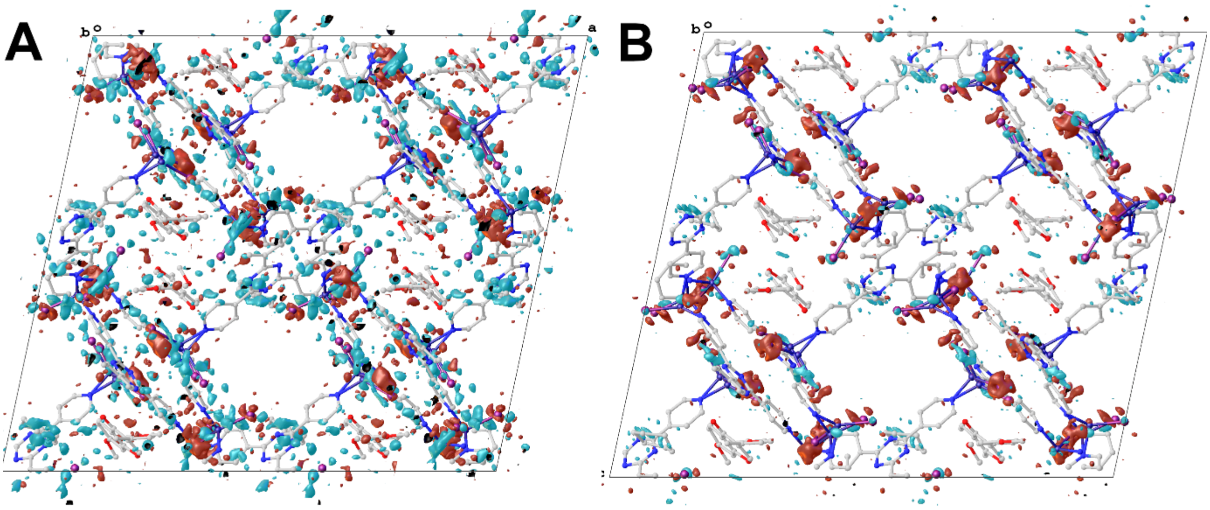

Figure S4 Residual electron density map (iso value: 1.0 e·Å^{-3}) for the measurements **2** for Cu Kα (**A**) and Cu Kβ (**B**) measurements with the sponge scaffold, analyte, and solvent in stick representation in b direction. H-atoms are omitted for clarity. Solvent masks were applied to the voids in both structures.

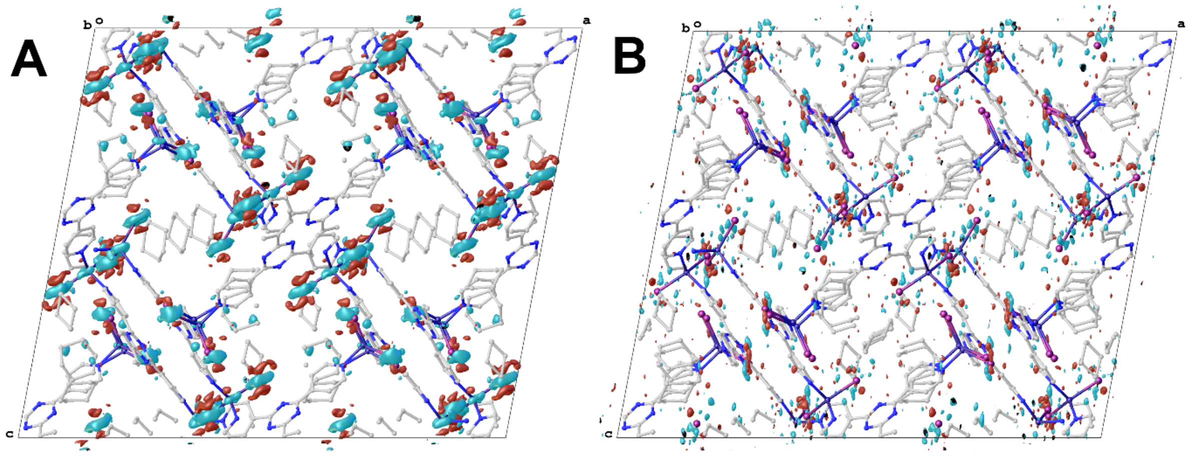

Figure S5 Residual electron density map (iso value: 1.2 e·Å^{-3}) for the measurements **3** for Cu Kα (**A**) and Cu Kβ (**B**) measurements with the sponge scaffold, analyte, and solvent in stick representation in b direction. H-atoms are omitted for clarity.

S5. Illustration of restrained solvent positions

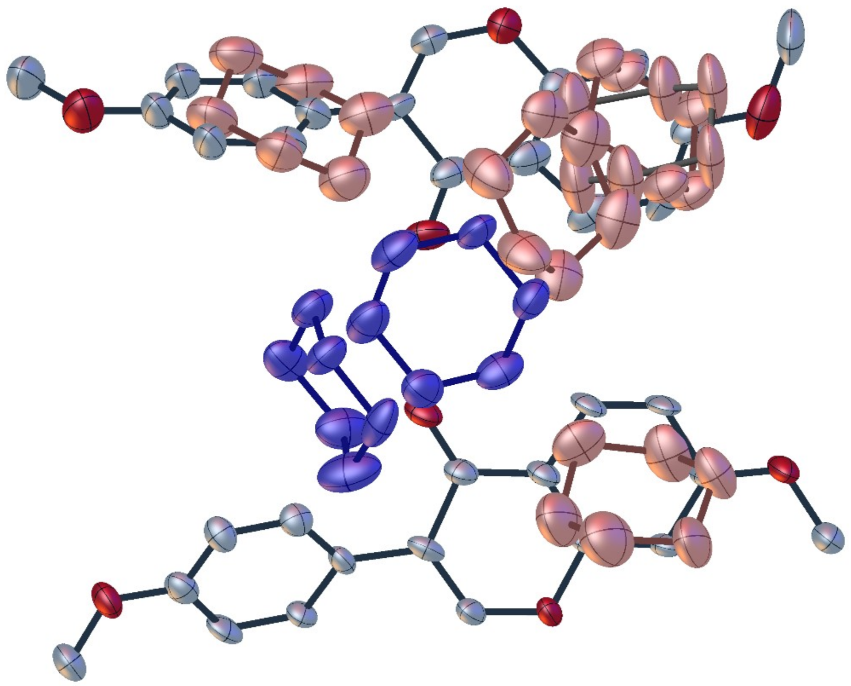

A

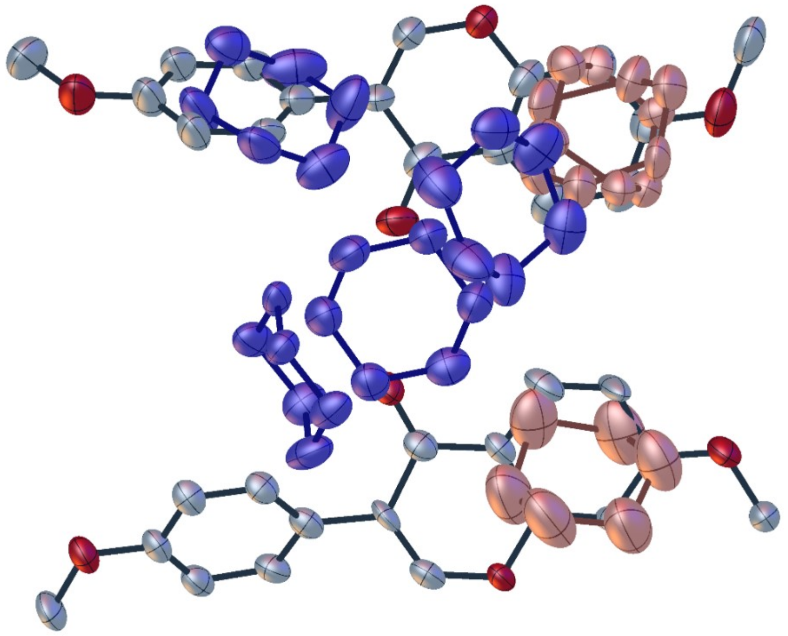

B

Figure S6: Contents of the asymmetric unit of measurements **1 a** (**A**) & **b** (**B**). Hydrogen atoms, as well as the scaffold has been omitted for clarity. Blue coloured units of Cy are freely refined without restrains or constrains on positional and occupancy parameters. Bronze coloured units have either restraints or constraints on positional and occupancy parameters. Red coloured atoms are oxygen atoms, differently coloured atoms are carbon atoms.
